# Supplementary material for: An ultra energy-efficient hardware platform for neuromorphic computing enabled by 2D-TMD tunnel-FETs
Source: Nat Commun. 2024 Apr 22;15:3392. doi: 10.1038/s41467-024-46397-3 (PMC11035659; doi:10.1038/s41467-024-46397-3)
Supplement: Supplementary file 1 — Supplementary Information [file 41467_2024_46397_MOESM1_ESM.pdf]

Supplementary Information

An Ultra Energy-Efficient Hardware Platform for Neuromorphic Computing Enabled by 2D-TMD Tunnel-FETs

*Arnab Pal[1], Zichun Chai[1], Junkai Jiang[1], Wei Cao[1], Mike Davies[2], Vivek De[2] and Kaustav Banerjee[1]**

[1]Department of Electrical and Computer Engineering, University of California, Santa Barbara, CA; [2]Intel Labs, Hillsboro, OR

*correspondence to: kaustav@ece.ucsb.edu

<u>Supplementary Note 1:</u> Introduction to Neuromorphic Computing

Since the discovery of MOSFET in the late 1950s, although the pace of development of semiconductor devices and computing architectures has been unrelenting, achieving computing efficiencies of the order of the human brain has been a distant dream. The human brain not only analyses data, makes decisions, communicates, but also maintains the vital functions of life all with a power budget of 20W, which is orders of magnitude lower than the estimated 1000 KW of power consumed by a state-of-the-art supercomputer accomplishing the same tasks **[1] (Supplementary Figure 1a)**. Moreover, the human brain is capable of accumulating experiences over its entire lifetime and can learn from only few mistakes, which is a far cry from the state-of-the-art machine learning algorithms that need a huge set of training data to be able to accomplish meaningful learning, and that too in a very specialized field. These drawbacks of conventional von Neumann (VN) computing point out to the need for developing emerging computing architectures, which closely mimic the biological brain, called neuromorphic (NM) computing, which was in fact conceptualized by Alan Turing in his seminal paper 'Intelligent Machinery' where the concept of a machine capable of being educated and trained was proposed **[2]**. However, even decades after the proposal, significant progress in the neuromorphic computing field was rather limited due to the inadequate understanding of neuroscience and immature device fabrication principles, until the proposal by Carver Mead in 1990 **[3]** where an analog circuit fabricated by transistors biased in the subthreshold region was shown to provide sufficient promise for mimicking human brain functionality on hardware. This led to a breakthrough in the NM computing field as the two components of any NM circuit – the neuron, responsible for information processing, and synapse – the communication and interface medium between two neurons, besides remembering and actively implementing the neural learning, could possibly be finally realized **(Supplementary Figure 1b)**. Furthermore, the synaptic learning rules, where neurons that fire together strengthen their neuronal connection, discovered by Hebb in the 1950s **[4]** were also discovered to be apt for implementing neuromorphic learning rules, and thus, a fully functional miniature brain could theoretically be realized. In this endeavor, several hardware implementations for neurons with different levels of abstraction in neuromorphic computing were proposed, among which the most promising are: biologically-plausible – which explicitly models the biological neurons and synapses, biologically-inspired – which aims to functionally replicate the biological nature of neurons and synapses, leaky-integrate-and-fire – a simpler hardware implementation of biologically-inspired neural networks but with less complexity, and McCulloch-Pitts – neuron models that are a simple derivative of the original McCulloch-Pitts neuron. However, although the biologically-plausible Hodgkin-Huxley neuronal models are the most biologically accurate, modeling the change of neuron membrane potential through ion flow across several dendritic channels, the sheer complexity of such a design makes hardware implementation unfeasible. In this aspect, the simpler leaky-integrate-fire neuronal model, which models the neuron as a single unit whose membrane potential is regulated by the receipt of incoming spikes **(Supplementary Figure 1c)**, strikes a good balance between implementation complexity and biological accuracy, and hence, is suitable for implementing artificial neurons in artificial neural nets as discussed later.

<u>*Biological Model:*</u> The biological neuron comprises of three parts: the dendrite – which collects the information from adjoining neurons; soma, the cell-body – where the processing takes place; and the axon – which transmits the action potential (electrical spikes) down to other neurons through respective synapses with discharge of neurotransmitters. Generally, the resting potential of a neuron is negative (around -70 mV) w.r.t its outside, and increases or decreases based on the excitatory or inhibitory neurotransmitters it receives from axons of other presynaptic neurons respectively. While reception of positively charged neurotransmitters like Na^+, K^+ and Ca^+ ions increase the neuron membrane potential and comprise the excitatory inputs, negatively charged ions like Cl^- are inhibitory and decrease the neuron membrane potential. The neuron subsequently fires or discharges similar excitatory/inhibitory neurotransmitters once its overall membrane potential increases to -50mV or above, and then resets to its resting potential again **(Supplementary Figure 1a)**. The firing of the neuron generates an electrical signal that travels down the axon into the axon terminals where it leads to the emission of neurotransmitters that eventually attach to the dendrites of the respective post-synaptic neurons (neurons which are driven by the presynaptic neurons) leading to enhancement/depression of their neuron membrane potential. Neuromorphic hardware, therefore, aims to mimic these exact biological processes in implementing both neuronal and synaptic behavior in a circuit configuration, analogous to the human brain, called artificial neural nets (ANNs) representing a collection of connected nodes and their interconnections, that loosely model artificial neurons and synapses **(Supplementary Figure 1b)**. These synapses are assigned a weight, which is representative of the neuronal connection strength and can be programmed through learning algorithms, which can either be supervised or unsupervised.

<u>*Generations of ANN networks:*</u> Traditionally, this hardware implementation of ANN has been achieved over three generations of ANN networks, which can be broadly classified based on the network topology and on the temporal resolution of the input signals. The first generation is based on McCulloch-Pitts neurons as the computational units **[5]**

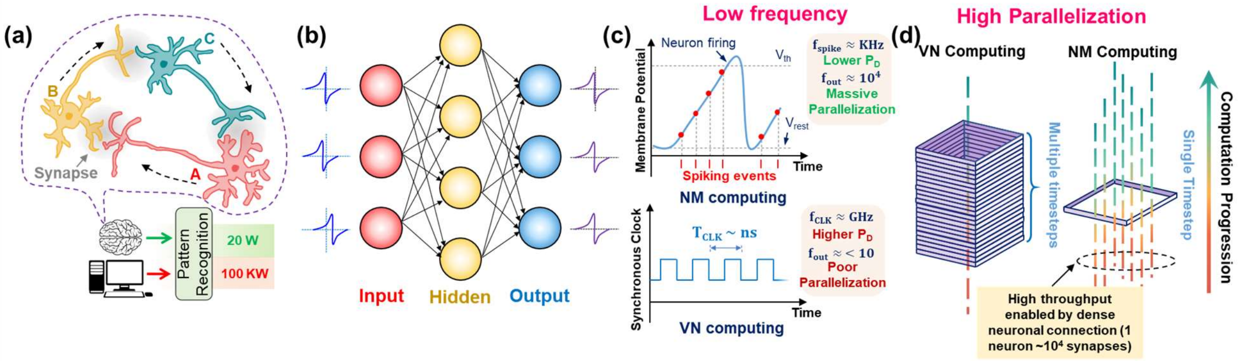

Supplementary Figure 1. Overview of Neuromorphic Computing. (a) Compares the power efficiency of the human brain against a conventional state-of-the-art von Neumann computer in pattern recognition. The figure on the top schematically illustrates the interconnections of the neurons and synapses, where the firing sequence of neuron A leads to firing of neuron B, and hence, neuron C. **(b)** Illustrates the feedforward network topology of a SNN where the signals in the time domain are input to the neurons of the input layer, which eventually are connected to the output layer through the hidden layer, where the synaptic weights are regulated by the learning rules. **(c)** Firing operation of a neuron (top) in KHz frequency vs the large ~GHz operational frequency of clock in VN computing. Membrane potential of the neuron increases upon receipt of every input spike, eventually leading to its firing once it exceeds the neuron firing threshold. **(d)** Large parallelization of NM computing w.r.t VN computing drives multiple computational steps in a single clock cycle, which is enabled by the large neuronal connection density in biological networks.

which are feed-forward neural nets, i.e., output does not flow back for error correction, and generally comprise very few hidden layers, i.e., layers present between the input – responsible for sampling the input signals, and the output – where the inference of the neural net is generated. This particular type of network topology, called perceptrons, can only generate digital outputs and have limited classification abilities (with low accuracy and a low subset of input combinations), it is not suitable for implementing learning algorithms, which therefore, make them unsuitable for practical purposes.

The second generation of neural nets, capable of operation with analog inputs/outputs and implementing learning algorithms based on gradient descent algorithms, such as back-propagation, are based on computational units that use activation function (sigmoid and tanh for example) for generation of possible output values. Such network topologies, therefore, require feedback for optimal learning, and are implemented on feedforward and recurrent neural nets. In addition, the analog output of these neural nets can be interpreted as the average firing rate of the biological neurons, with an output of 1 representing the maximum achievable biological neuronal spike efficacy. However, although such a model for computation based on the firing rate of neurons is biologically more plausible w.r.t first-generation of ANN, it is not biologically accurate, since, while in biology decision making or inferencing can take place in as little as few spikes, for the firing rate model to work in practice, multiple neuron spikes over a long period must be sampled to build up a robust model for firing rate. This limits both processing capabilities and degrades energy efficiency **[5]**. For the hardware to mimic a human brain therefore, the biological plausibility of artificial neurons and synapses must be improved, which can be achieved by implementing a hardware model that operates on the spikes received, instead of the spiking frequency, and such a network represents a spiking neural net (SNN) **(Supplementary Figure 1b)**, which is in fact, the third generation of ANN networks. Besides modeling the biological plausibility, such ANN networks also model both excitatory or inhibitory pulse inputs, and neuronal membrane leakage that represents the slow decay of membrane potential over time upon firing inactivity.

Advantages of SNN: This biological resemblance of SNN networks results in massive parallelism (due to one neuron being connected to many neurons through synapses) **(Supplementary Figure 1d)**, potential for online learning and event-learning processing, along with fast inference and low-energy computation **(Supplementary Figure 1c)**. Moreover, this also results in SNN networks being asynchronous, independent of a common clock signal since they work on spike-based signals instead of a continuous valued signal input in other ANNs. This asynchronicity of the SNN circuits, decoupling the spiking operation of one neuron independent w.r.t the others, along with biologically inspired low neuronal spiking frequencies, makes such a SNN circuit very low energy consuming since they dissipate less energy intensive leakage power over the course of their operation duration. This results in a significant benefit for efficient hardware implementation over conventional ANN, where ANN's large requirement for computing energy limits practicality as need for energy-efficient and computing-intensive computing grows, especially in handheld processing devices in this age of big-data. In addition, SNN networks with asynchronous mode of computation along with event-based sensing, have been shown to be able to perform pseudo-simultaneous information processing **[6],** which enables an immediate generation of an approximate output upon receipt of the first input spikes, in contrast to conventional ANN networks where all layers need to be fully updated before generation of an output. This, therefore, also helps in

minimizing computation time. Therefore, in light of these benefits of SNN networks in achieving biological plausibility and low-energy computing, they are suitable for implementing an artificial brain with hardware and have been the focus of recent commercial NM chips – Intel's Loihi **[7]** and IBM's TrueNorth **[8]**.

<u>Supplementary Note 2:</u> Introduction to TFET

Motivation for steep-subthreshold slope devices: Lowering the power consumption of electronic devices is critical to achieving energy-efficient computing, especially in the age of big-data where an exponential increase in computing demands an equally exponential increase in the energy needed for data processing. This is not only important for improving overall efficiency of data processing, but also to repel the imminent threat of global warming through energy-intensive inefficient computing. Between the two sources of power dissipation (static and dynamic) possible in any chip, reduction in both can be achieved by minimizing the operational (or supply) voltage (V_{DD}) (**Supplementary Figure 2**). This is because, while a transistor operating at smaller V_{DD} not only dissipates a lower OFF-current (through smaller parasitic leakage mechanisms of gate induced drain lowering and drain induced barrier lowering), but the smaller V_{DD} also helps in lowering the static power (a product of OFF current and V_{DD}). Moreover, an even stronger reduction in the dynamic power component ($\propto V_{DD}^2$) is obtained at a lower V_{DD} due to its quadratic dependence on V_{DD}. This, therefore, makes the reduction in V_{DD} a primary knob to reduce the overall power density of the chip, provided the transistor can be operated with a minimum ON-OFF current ratio. However, since this reduction in V_{DD} with a minimum ON-OFF ratio is only possible in a transistor with steep current-voltage (or turn-on) characteristics, i.e., low subthreshold swing (*SS*), such a device in indispensable for low-energy computing.

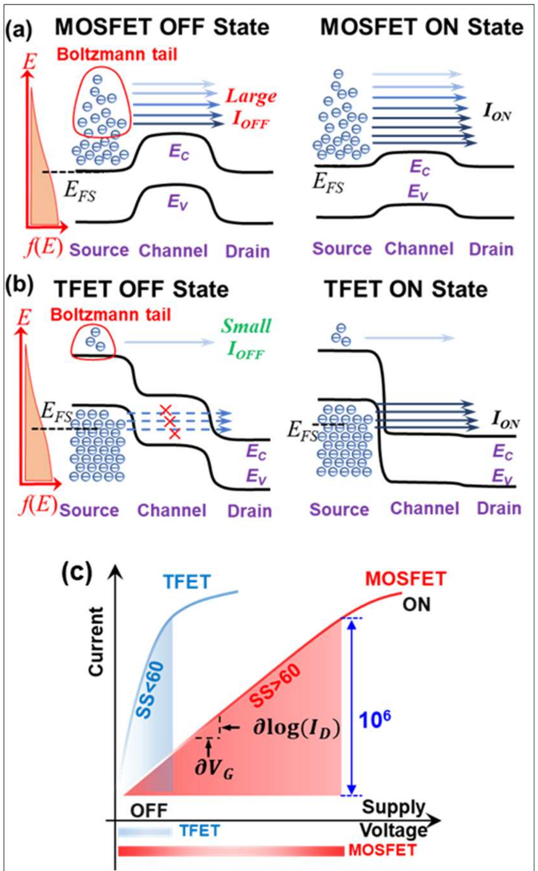

Supplementary Figure 2. Comparison of band-diagrams of MOSFET against TFET under their OFF- and ON-conditions. While the thermionic barrier limited transport in **(a)** MOSFETs lead to larger ON-current w.r.t the tunneling limited carrier transport in **(b)** TFETs, the bandgap filtering of high-energy carriers in the TFET significantly reduces its OFF-current. Moreover, the sudden alignment of the TFET channel conduction band with its source valence band under the application of V_{GS} leads to a steep increase of its ON-current, leading to low *SS*. **(c)** Comparison of the current (I_D)-voltage (V_G) characteristics of a MOSFET with a TFET, demonstrating why the low *SS* characteristics of the latter allow the operational voltage to be scaled down without sacrificing the ON-OFF ratio.

TFET over other low-SS devices: Therefore, although we desire a transistor that can be turned OFF and ON with the application of a miniscule V_{DD} (with a fixed ON-OFF ratio), the ubiquitous electronic switch, the MOSFET, is characterized with a physically limited minimum subthreshold swing (inverse of subthreshold slope) of 60 mV/decade at room temperature (i.e., transistor drain current can be increased by 10x with the application of 60 mV gate voltage), which ultimately limits the minimum operational voltage to 360 mV for a ON-OFF ratio of 6 decades **(Supplementary Figure 2)**. Overcoming this fundamental barrier, therefore, has become the primary interest for device engineers over the last several decades, and among the several solutions proposed, the notable ones are: tunneling-FETs (TFETs), negative-capacitance FETs (NCFETs), Dirac-source FETs (DSFETs), impact-ionization FETs (IFETs), and nano-electro-mechanical FETs (NEMFETs). However, despite the theoretical promise, realistically there are several design and reliability concerns that have hampered their practical application. For example, NCFETs suffer from severely limited design space constraints that hamper its practical realization **[9]**, in addition to enhanced hysteresis and reliability issues; DSFETs with low source DOS limit maximum achievable ON-current and yield a *SS* not lower than its contemporaries **[10]**, and IFETs and NEMFETs suffer from severe reliability issues due to continuous generation of electron-hole pairs **[11]** and mechanical failure of the conducting cantilever **[12]**, respectively. Therefore, despite limitations of unidirectional current flow (since source and drain terminals are not interchangeable, unlike in MOSFETs), low ON-current, and large gate-drain Miller capacitance, TFETs are more practical for realizing low-energy low-frequency circuits as described in the main article.

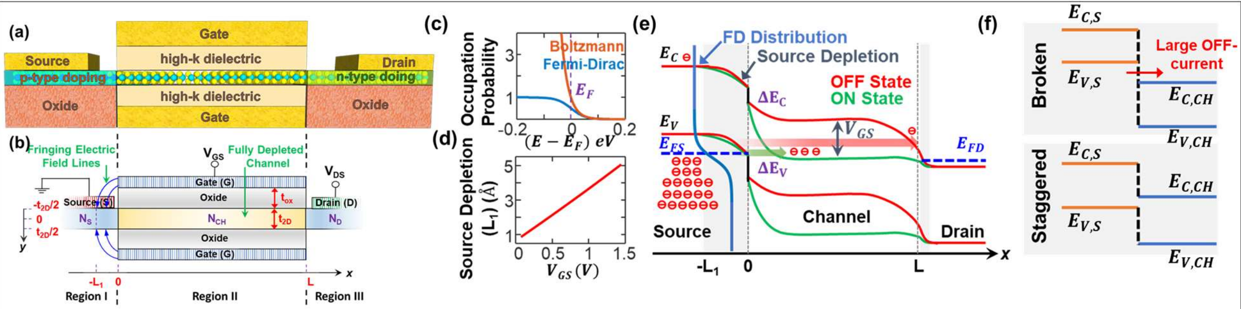

Supplementary Figure 3. TFET cross-section and physics. (a) Cross sectional view of the double-gate 2D n-TFET. **(b)** Cross section of the device under operation at a certain bias showing the fringing electric field lines emanating from the gate and terminating into the source, the fully depleted channel, and the profile of the source depletion region. The channel length of the device is L, L_1 refers to the source depletion length, t_{2D} is the thickness of the 2D channel, while t_{ox} is the gate oxide thickness. The source, channel and drain doping are denoted by N_S, N_{CH}, and N_D respectively. The source is grounded, and a voltage of V_{GS} and V_{DS} are applied to the gate and drain terminals, respectively. **(c)** Comparison of the Fermi-Dirac (FD) and Boltzmann distribution as a function of energy, showing the inaccuracy of the latter (occupation probability > 1) in estimating carrier occupation probabilities at energies below the Fermi level. **(d)** Evaluation of the source depletion length as a function of the applied V_{GS}, showing a linear increase with an increase of the V_{GS} which happens due to increased electric field lines. **(e)** Sketch of the energy bands [conduction band (E_C), valence band (E_V)], source (E_{FS}) and drain (E_{FD}) Fermi levels, and the OFF- and ON-state of the device under the application of a certain V_{GS}. The application of the V_{GS} causes a sudden turn-on of the device due to the sudden alignment of the channel conduction band with the source valence bands. **(f)** Schematic of the broken and the staggered band-alignments at a heterojunction. While both staggered and broken band-alignments enhance the ON-current of the TFET, the omnipresent overlap of the channel conduction band with the source valence band in the broken band heterostructure, even without the application of any V_{GS}, increases the device OFF-current, degrading energy-efficiency.

Working principle of a TFET: TFETs work on the principle of band-to-band-tunneling (BTBT) of carriers, where carriers (electrons in a n-TFET), upon the application of a suitable gate-source voltage, tunnel from the filled source valence band to the empty channel conduction band, constituting in a sudden increase of their ON-current, thereby resulting in low-*SS* (**Supplementary Figure 2**). Fundamentally, the *SS* of a transistor, describing the steepness of its switching from ON to OFF-states can be described as:

$$SS = \frac{\partial V_{GS}}{\partial(\log_{10} I_{DS})} = \frac{\partial V_{GS}}{\partial \psi} . \frac{\partial \psi}{\partial(\log_{10} I_{DS})} \quad (1)$$

where V_{GS} is the gate-source voltage, ψ is the channel-surface potential, and I_{DS} is the drain-source current. The first term in the RHS of **Supplementary Equation 1** represents the ease of modulation of the channel surface potential upon the application of the gate-source voltage and is representative of the efficacy of the transistor electrostatics, while the second term determines the rate of change of the transistor drain current with the corresponding change in the surface potential and is determined by the physics of carrier transport. In this aspect, the adoption of 2D-materials with their defect free interface **[13][14][1][15]**, low trap states and inherent thinness only enhances the electrostatics, thereby tending the first parameter to its minimum value of unity (a particular change in V_{GS} leads to an identical change in ψ), while the BTBT transport property of the TFET reduces the second factor, i.e., a unit change in ψ leads to a larger change in I_{DS} w.r.t to what is achieved with MOSFETs (carrier transport over the thermionic barrier).

Low OFF- and ON-current in TFETs: **Supplementary Figure 2** compares the band-diagram of a n-TFET against a n-MOSFET under both OFF and ON conditions **[1]**. As observed, under the OFF condition, both n-TFET and the n-MOSFET present large energy barriers for electrons in the source to transport across the channel into the drain. While in a MOSFET, the energy barrier arises from the offset in the conduction band in the source and channel (due to difference in their doping concentration and applied gate-source bias), with only carriers having an energy higher than the barrier being able to transport, the barrier in the TFET is effectively the bandgap of the channel (for a homojunction), thereby presenting an effectively larger barrier. Moreover, since in such a scenario only carriers at the top of the source conduction band of the TFET can flow into the drain constituting a drain current, the dramatic decrease of the carrier occupation due to bandgap filtering (carrier occupation almost exponentially decreases above the Fermi level energy due to Fermi-Dirac statistics, and density of states in bandgap is zero) significantly reduces the OFF-current w.r.t the MOSFET, leading to significant energy savings.

In the ON state, although the channel bands in both n-MOSFET and n-TFET are lowered upon application of a positive V_{GS}, the mechanism for carrier flow is different in the two devices. While in a TFET the lowering of the channel conduction band w.r.t the source valence band opens up a tunneling window for carriers to tunnel from source to channel across a tunnel barrier whose height is equal to the channel bandgap (for a homojunction; tunneling barrier width is a function of the applied bias and the doping, and the ON-current increases as the tunneling barrier width decreases), in a MOSFET this constitutes a simple reduction in the energy barrier for electrons in the source. Therefore, although this transport phenomena in a TFET results in a low *SS*, the consequence for the carriers to tunnel across a barrier (with probability < 1) results in a smaller ON-current w.r.t MOSFET, where carriers diffusing over the energy barrier (with

probability = 1) instead of through it presents minimal resistance. Since tunneling probability of carriers determine the ON-current of the TFET, ways to enhance the ON-current while simultaneously minimizing OFF-current and yielding a low-*SS* must be explored. The next section discusses the TFET geometry used for the simulations in this paper and explains the device operation in more detail.

<u>Supplementary Note 3:</u> TFET Cross-Section and Material-Combination

Need for source-channel heterojunction: The need for maximizing the ON-current with simultaneous reduction in both the OFF-current and the *SS* in a TFET **(Supplementary Figure 3)** necessitates the choice for a suitable source-channel material combination with suitable band alignment **(Supplementary Figure 3f)** that can deliver a low effective tunnel barrier during band-to-band tunneling. This, therefore, requires that both source conduction and valence bands be higher in energy w.r.t the corresponding channel energy bands thereby not only minimizing the effective tunnel barrier (difference between the valence band edge of the source and the conduction band edge of the channel, i.e., $E_{\mathrm{C,CH}} - E_{\mathrm{V,S}}$), but also leading to an effective increase in the tunneling electric field. This can be achieved with a staggered source-channel band alignment **[16]**. In this regard, the source-channel material combination of WTe_2-MoS_2 identified in **[16]** and modeled in **[17]** provide a high ON-current of 728 μA/μm with a minimal 4-decade average *SS* of 18 mV/decade, and thus, becomes the material combination of choice.

Consideration of important device physics: The 2D-semiconductor-channel double-gate (DG) n-type TFET device under consideration in this work is shown in **Supplementary Figure 3a**, and the device cross-section under a certain bias is shown in **Supplementary Figure 3b [17]**. Since the TFET is a n-type TFET, turning ON under the application of a positive gate bias, the source is heavily p-doped, the channel is intrinsic or slightly n-doped, and the drain n-doped. Under an application of a positive gate bias, the channel can be assumed to be fully depleted (Region II) while a part of the p-type source is also depleted (Region I) due to the electric field lines emanating from the gate and terminating into the source. Since the source in TFETs are heavily doped to maximize performance, the Fermi level generally lies within the conduction/valence bands depending on a p-/n-type TFET, respectively, thereby necessitating the importance of incorporating the accurate Fermi-Dirac statistics in carrier distribution modeling **(Supplementary Figure 3c)**. Although the heavy doping in the source reduces the depletion length **(Supplementary Figure 3d)**, the length of the source depletion region is still important to consider because it heavily regulates the ON-current of the device through modulation of the effective tunneling width, i.e., the tunneling electric field. **Supplementary Figure 3e** shows the operation of the device under both the OFF- and the ON-state, where the positive gate bias of the device in its ON-state lowers the channel conduction band below the source valence band, thereby allowing appreciable number of electrons to tunnel to the channel conduction band from the source valence band and constituting a forward current. Similarly, carriers from the drain electrode move through the channel to tunnel into the source across the tunneling barrier, thereby constituting a reverse current. The net current in the device at any bias of operation is therefore, given by the difference of these two currents. Further details on the device physics and the compact model of the device can be found in **[17]**.

<u>Supplementary Note 4:</u> Comparison of I_{DS}-V_{DS} and I_{DS}-V_{GS} Characteristics

The I_{DS}-V_{DS} and the I_{DS}-V_{GS} characteristics of the similarly sized (similar effective channel width and channel length) 2D-TFET and the 7 nm technology node LSTP PTM model have been compared in **Supplementary Figure 4a**, **Supplementary Figure 4b**, and **Supplementary Figure 4c**, where V_{DS} is the applied drain-source bias. As seen from **Supplementary Figure 4a**, the ON-current of the LSTP model is markedly higher than that of the 2D-TFET model, however, its ON/OFF ratio of around 7 orders of magnitude is smaller than that of 11 orders in the latter, which is because of the low *SS* and low-OFF current that the TFET offers. This has huge ramifications in the final figure of merit in the energy consumption of the neuromorphic circuit, as shown in **Figure 6** of the main article, where the low SS of the 2D-TFET delivers close to 100-folds improvement in the energy-efficiency

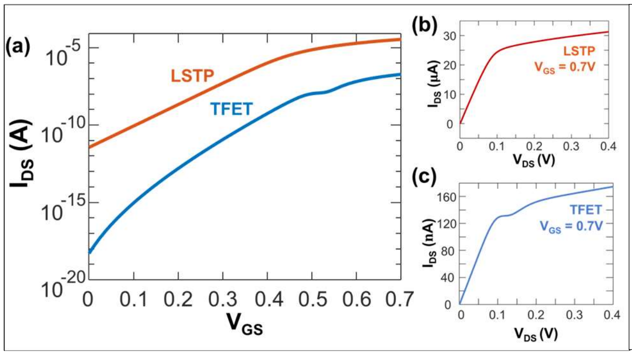

Supplementary Figure 4. 2D-TFET vs MOSFET I_{DS}-V_{GS} comparison (a) I_{DS}-V_{GS} comparison of the 2D-TFET and the LSTP model used in the circuit simulation, sized appropriately to deliver equal device capacitance and channel length (11 nm). LSTP FinFET model uses one finger for a total effective width of 42.5 nm, and DG-2D-TFET uses a channel width of 21.25 nm for an effective width of 42.5nm. I_{DS}-V_{DS} comparison of the **(b)** LSTP and **(c)** 2D-TFET models simulated at V_{GS} = 0.7V.

of the neuromorphic circuit compared to that of the LSTP implementation. Also, the 11 nm channel length TFET delivers respectable drain current saturation with V_{DS} (**Supplementary Figure 4c**), thereby delivering good gain in the digital inverter logic (**Figure 2** of the main manuscript), which ultimately leads to good noise margins in SRAM design (**Figure 2** of the main article) and delivers lower short-circuit power dissipation compared to implementations with FinFET models.

<u>Supplementary Note 5:</u> Hardware Implementation of a Full-Adder and Up-Counter

A. <u>Implementation of a Full-Adder:</u>

A full adder is a digital circuit that performs the addition of two digital inputs (A and B) with an additional input carry (C_{in}). The truth table for such a full adder circuit generating sum output of S and carry output of C_{out} therefore, is given as:

A	B	C_{in}	S	C_{out}
0	0	0	0	0
0	0	1	1	0
0	1	0	1	0
0	1	1	0	1
1	0	0	1	0
1	0	1	0	1
1	1	0	0	1
1	1	1	1	1

Therefore, the logic expressions for the sum and carry out of such a full adder is given as:

$$S = A \oplus B \oplus C_{\text{in}}$$

$$C_{\text{out}} = (A.B) + \left(C_{\text{in}}.(A \oplus B)\right)$$

Hence, the circuit for implementing such a full-adder functionality can be achieved through the use of 2-input XOR gates, 2-input AND gates and 2-input OR gates. The circuit implementation, therefore, is:

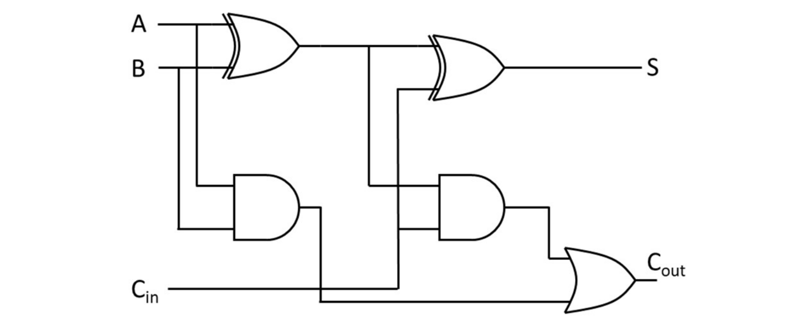

Supplementary Figure 5. Full-adder circuit. Figure shows a full-adder circuit implemented with XOR, AND, and OR gates, generating sum output S, and output carry C_{out}.

The circuit described in **Supplementary Figure 5** achieves addition of two single bit signals (A and B) with a single bit input carry of C_{in}. Achieving multi-bit addition, therefore, necessitates the cascading of several FA circuits (the number of such FA circuits equals the number of bits to be added) where the output carry of the first FA (implementing

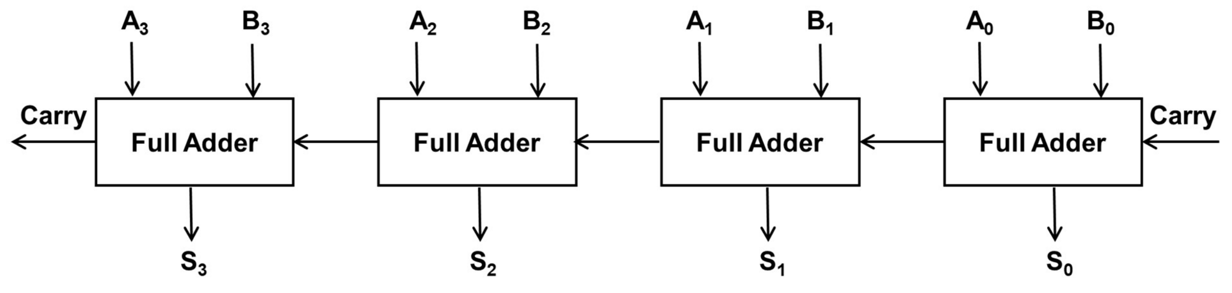

Supplementary Figure 6. Schematic of a 4-bit full adder. 4-bit full-adder circuit is implemented by connecting the output carry of one FA to the input carry of the other. The generated sum bits ($S_3S_2S_1S_0$) result from the addition of inputs A and B.

least significant bit) feeds into the input carry for the next FA circuit. Therefore, the circuit responsible for adding two 4-input signals *A* ($A_3A_2A_1A_0$ are the individual bits) and *B* ($B_3B_2B_1B_0$ are the individual bits) with an input carry of C_{in} is as shown in **Supplementary Figure 6**.

B. Implementation of an Up-Counter:

A digital up-counter counts up every clock cycle from a minimum value of binary 0 to a maximum value achievable by that multi-bit input. For example, a 4-bit digital counter with output bits of $Q_3Q_2Q_1Q_0$ counts up from a value of $(0000)_2$ to $(1111)_2$ through the following sequence:

Q_3	Q_2	Q_1	Q_0
0	0	0	0
0	0	0	1
0	0	1	0
0	0	1	1
0	1	0	0
0	1	0	1
0	1	1	0
0	1	1	1
1	0	0	0
1	0	0	1
1	0	1	0
1	0	1	1
1	1	0	0
1	1	0	1
1	1	1	0
1	1	1	1

Among the several ways for implementing such a 4-bit up-counter circuitry, the most convenient way is through implementation with JK flip-flops. The truth table for a single-bit JK flip-flop is:

J	*K*	Q_{n+1}	State
0	0	Q_n	Hold
0	1	0	Reset
1	0	1	Set
1	1	$\overline{Q_n}$	Toggle

Where *J* and *K* are the 2-inputs, Q_{n+1} is the output of the flip-flop at the next clock cycle, and Q_n is the current output of the flip flop. Therefore, as clearly seen from the preceding truth table, the output of the JK flip-flop toggles every clock cycle when the inputs to J and K are 1 each. Therefore, keeping this in mind, it is easy to see that a JK flip-flop with both of its inputs shorted to 1 would implement the functionality of the bit Q_0 of the up-counter, since it toggles every clock cycle. Similarly, the bit Q_1 toggles whenever the Q_0 is equal to 1, otherwise it holds its prior state of bit 0. Therefore, to implement the functionality of bit Q_1, bit Q_0 must be connected to both the inputs of the JK FF implementing Q_1. Similarly, bit Q_2 only toggles when both bits of Q_0 and Q_1 are equal to 1, it holds its prior state of 0 otherwise. Hence, to implement the functionality of bit Q_2, the AND product of bits Q_0 and Q_1 must be shorted to both the inputs of the JK FF implementing Q_2. Finally, following the same logic, the AND product of bits Q_0, Q_1 and Q_2 must be shorted to the inputs of the JK FF implementing Q_3. This completes the design of the up-counter as shown in

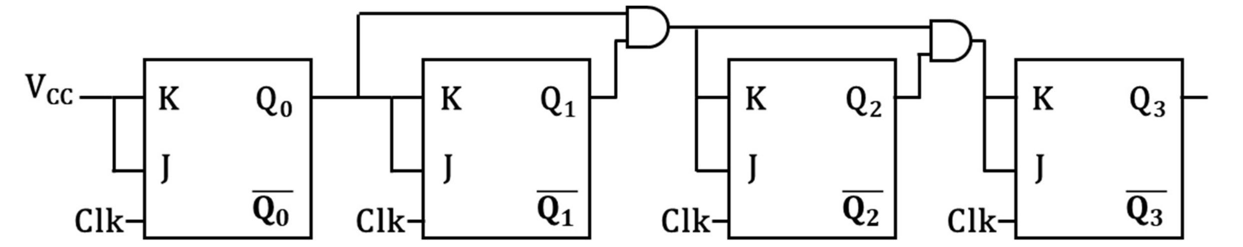

Supplementary Figure 7. Schematic of a 4-bit up-counter. 4-bit up-counter (output of $Q_3Q_2Q_1Q_0$) counting up from a value of $(0000)_2$ to $(1111)_2$ once every clock cycle is implemented with JK flip-flops with inputs (JK) shorted such that the output state of the JK FF toggles every clock cycle. The input for the JK FF implementing the output bit of Q_2 is an AND product of the two output bits of Q_0 and Q_1, while that for the JK FF implementing Q_3 is an AND product of Q_0, Q_1 and Q_2.

Supplementary Figure 7. In the actual circuit implementation, a reset functionality has been added to the JK FFs for clearing their outputs whenever that reset signal is asserted. The reset signal is eventually connected to the output of the comparator in **Figure 3** of the main article, such that every neuron fire resets the up-counter. Furthermore, since the up-counter in **Figure 3** of the main article is only meant to count (inputs to J and $K = 1$) only when the neuron is not firing (i.e., output of the comparator is low), therefore, the inverted output of the comparator (implemented through the inverter in red in LIF circuit following the comparator) is connected to the terminal J of the first JK FF implementing the functionality of bit Q_0 (**Figure 3** of the main article).

<u>Supplementary Note 6:</u> Use of 1's and 2's complement in binary subtraction:

Subtraction operation in digital circuits, resulting in the subtraction of the subtrahend (B) from the minuend (A), i.e., implementing $Y = A - B$, where Y is the output, is accomplished by an operation very similar to the adder operation except that the 2's complement of the subtrahend (B) is added to the minuend (A). The resulting operation generates an output and an additional carry bit which is 1 when the difference is positive, or 0 when the difference is negative. This section describes such an operation in detail, where a 5-bit signal 'B' is subtracted from a 5-bit signal 'A'.

Consider A = 10010, i.e., $(18)_{10}$ and B = 01000, i.e. $(8)_{10}$, thereby resulting in $Y = A - B$ = 01010, i.e. $(10)_{10}$. To achieve such a subtraction operation of the subtrahend B from the minuend A, the following steps are carried out in order:

1. *Step – 1: Evaluation of the 1's complement of subtrahend B*: The 1's complement of any digital bit is given by the complements of the individual bits. Therefore, the 1's complement of B is:

   B = 0 1 0 0 0
   1's complement = 1 0 1 1 1

2. *Step – 2: Evaluation of the 2's complement of the subtrahend B:* The 2's complement of any digital bit is obtained by adding binary 1 to the 1's complement. Therefore, the 2's complement of B is:

   1 0 1 1 1
   \+ 0 0 0 0 1
   2's complement = 1 1 0 0 0

3. *Step – 3: Addition of the 2's complement of B to A:* The 2's complement of the subtrahend B is finally added to the minuend A to evaluate $Y = A - B$:

   A = 1 0 0 1 0
   2's complement of B = + 1 1 0 0 0
   Y = 1 0 1 0 1 0

4. *Step – 4: Carry bit:* Since the carry bit of the resulting operation in step 3 is 1, therefore, the difference is positive and we can disregard the sign bit to get the value of the difference. Hence, the result of the subtraction of B from A is Y = 01010 = $(10)_{10}$ which is the value expected.

   If the carry bit is 0 however, the difference is negative, and the value of the difference is then obtained by evaluating the 2's complement of the output.

<u>*Use of subtractor circuit for Synapse weight calculation:*</u> A subtractor circuit can help evaluate the synaptic weight between two neurons. Let us assume two firing neurons – one presynaptic neuron, and one postsynaptic neuron with distinct firing events of t_{PRE} and t_{POST} (evaluated at a measurement time t), respectively, where the firing event of the presynaptic neuron is assumed to drive the firing event of the postsynaptic neuron. Since the counter value of the JK

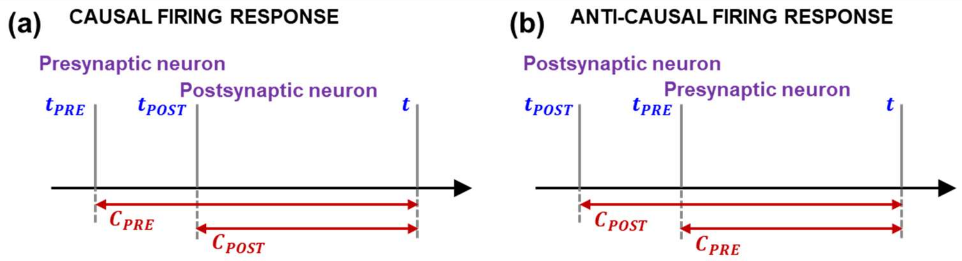

Supplementary Figure 8. Causal and anti-causal firing events. Schematic shows the firing events of the **(a)** presynaptic and **(b)** postsynaptic neuron firing events. t_{PRE} and t_{POST} refer to the firing events of the presynaptic and postsynaptic neurons, measured at a time t; while C_{PRE} and C_{POST} are the counter values of the neurons, respectively. For $C_{\mathrm{PRE}} > C_{\mathrm{POST}}$ the firing behavior is causal (increase in synaptic weight), otherwise, it is anti-causal (decrease in synaptic weight).

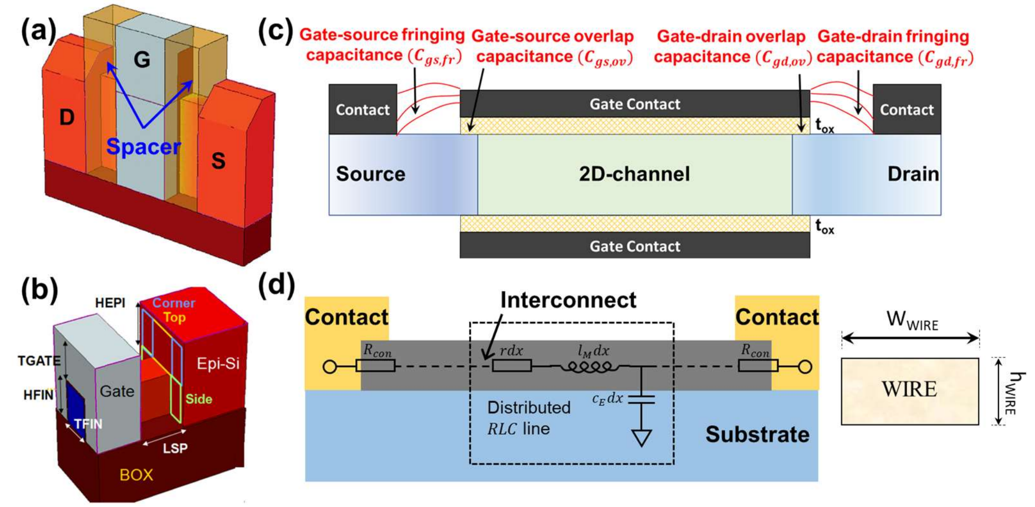

Supplementary Figure 9. Device and Interconnect capacitance models. (a) Schematic of the FinFET device showing Source (*S*), Drain (*D*) and Gate (*G*). The channel extends from the *S* to the *D* and is surrounded by the *G* from three sides. Nitride spacers separating the gate from the source and the drain are also shown. **(b)** Cross-section of the FinFET device showing half of the channel and the source/drain electrode. **(c)** Cross-section of the 2D-TFET channel showing the parasitic gate-source/drain fringing electric field lines contributing to the fringing capacitance ($C_{gs,fr}$/$C_{gd,fr}$), and the gate-source/drain overlap ($C_{gs,ov}$/$C_{gd,ov}$) capacitance. **(d)** Parasitics in an interconnect showing capacitive, inductive, and resistive components. The cross section of the interconnect and the wire width (w_{wire}) and height (h_{wire}) are shown to the right. R_{con} refers to the contact resistance of the interconnect to the S/D metal, r is the resistance per unit length of the wire, l_M is the magnetic-components of the inductance, and c_E is the electrical-capacitance.

FF of the learning circuit of a neuron (**Figure 3** of the main article) resets every time the neuron fires, therefore, the counter value of the neurons at a particular time t yields the information about the number of clock cycles elapsed since their last firing event. Assuming measured counter values of C_{PRE} and C_{POST} for the pre- and post-synaptic neurons, respectively, a larger value of C_{PRE} implies that the presynaptic neuron fired before the firing event of the postsynaptic neuron, as shown in **Supplementary Figure 8a**. This refers to a causal firing event since the firing event of the presynaptic neuron can be assumed to be driving the firing event of the postsynaptic neuron. For example, considering C_{PRE} = 20 and C_{POST} = 15, the difference of their counter values, i.e., 5 (evaluated in the learning subtractor circuit of **Figure 3** of the main article), implies that the presynaptic neuron fired 5 clock cycles before the firing event of the postsynaptic neuron. This results in a positive synaptic weight, with the synaptic weight increasing as the firing events tend to be closer (i.e., a smaller positive difference in the counter values). For an anti-causal firing behavior however, i.e., when C_{PRE} is smaller than C_{POST} (**Supplementary Figure 8b**), the postsynaptic neuron fired before the firing of the presynaptic neuron, resulting in a negative counter difference (-5). This implies a decrease in the synaptic weight between the two neurons, with a smaller negative counter difference implying a stronger decrease in the synaptic weight.

Supplementary Note 7: Device and Interconnect Capacitance Extraction

Device and Interconnect Models: This section explores the device and interconnect models used to model the associated device **[16][17]** and interconnect **[18]** parasitics in the designed neuromorphic circuit, followed by the capacitance extraction in the entire neuromorphic circuit. The schematic of a typical commercial FinFET transistor is shown in **Supplementary Figure 9a**, showing the source, channel, and drain regions, with gate oxide dielectric and the nitride spacer for reducing the gate-source and gate-drain interference. The detailed cross-sectional view of the FinFET channel is shown in **Supplementary Figure 9b** showing the related feature sizes of the FinFET. The *LSP* denotes the length of the source-channel underlap, *TGATE* shows the thickness of the gate metal, *HFIN* and *TFIN* are the height and thickness of the fin, respectively. Therefore, the effective width (W_{eff}) of the FinFET is given as: $W_{\mathrm{eff}} = 2 \times H_{\mathrm{fin}} + T_{\mathrm{fin}}$. For the 7nm FinFET model **[19]**, $T_{\mathrm{fin}} = 6.5$ nm, $H_{\mathrm{fin}} = 18$ nm thereby leading to $W_{\mathrm{eff}} = 42.5$ nm, with a channel length of $L_{\mathrm{ch}} = 11$ nm. The parasitic capacitances between the gate and the source/drain are comprised of two components – the overlap ($C_{\mathrm{gs,ov}}/C_{\mathrm{gd,ov}}$) and the fringing ($C_{\mathrm{gs,fr}}/C_{\mathrm{gd,fr}}$) capacitance between the gate electrode and the source/drain extension/electrode region, respectively. In accordance with the technology file for the 7nm LSTP FinFET model, $C_{\mathrm{gs,ov}} = C_{\mathrm{gd,ov}} = 25.6$ pF/m, and $C_{\mathrm{gs,fr}} = C_{\mathrm{gd,fr}} = 25$ pF/m, thereby leading to a net gate-source/drain overlap and fringing capacitance of 1.088 aF and 1.0625 aF, respectively, for an effective channel width

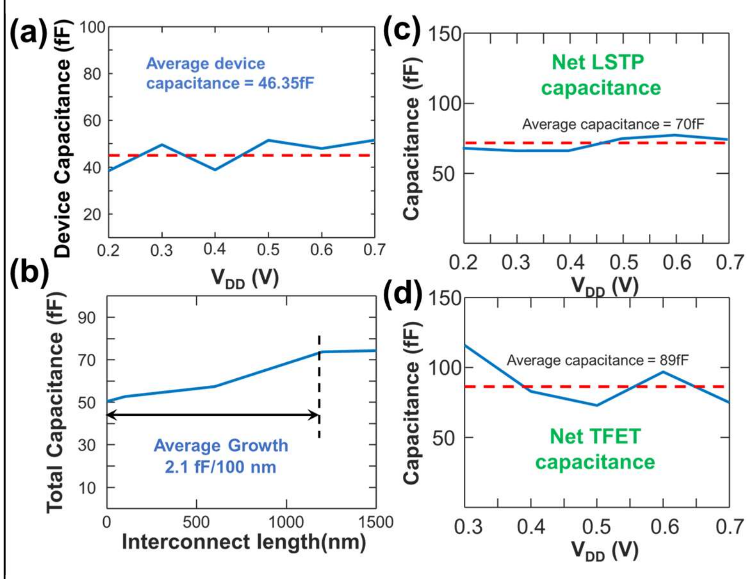

Supplementary Figure 10. Extracted device and circuit capacitances. (a) Average extracted device capacitance of the LSTP implemented NM circuit across a V_{DD} range of 0.2-0.7V showing an average value of 46 fF. Minor variations in the data are due to extraction errors. **(b)** Simulation of the net (device plus interconnect) capacitance of the circuit as the interconnect length is increased showing a roughly linear variation. The interconnect length refers to the length of the interconnect, which is connected between adjacent logic blocks, and is the same for all the interconnects used in the circuit. An average growth of the interconnect capacitance at 2.1 fF/100 nm is observed over the marked region. **(c)** Extraction of the total circuit capacitance in the LSTP implementation as a function of the V_{DD} ranging from 0.2V to 0.7V. A 1000 nm of interconnect length has been assumed to be present between each successive logic blocks, thereby yielding an average interconnect capacitance of 24 fF, around half of that of the device capacitance of 46 fF, for a total capacitance of ~70 fF. **(d)** The capacitance of the 2D-TFET circuit implemented with 1000 nm of interconnect capacitance, yielding a slightly higher capacitance of 89 fF due to overestimation at 0.3V V_{DD}. This happens because of extraction issues at lower frequencies (f_{max} at 0.3V is 700 Hz).

of 42.5 nm. Therefore, the entire gate-source and gate-drain parasitic capacitance is given by the summation of these two parallel capacitances, and is equal to $C_{\mathrm{gs,parasitic}} = C_{\mathrm{gd,parasitic}} = 2.15$ aF. **Supplementary Figure 9c** shows the cross-section of the modeled 2D-TFET and the associated gate-source/drain overlap and parasitic capacitances. Since the simulated TFET in our model is a double-gate device, therefore, to yield an effective channel width of 42.5 nm, we need an actual device width of half of that, i.e., 21.25 nm. Moreover, the extreme thinness of the 2D-body minimizes any source-channel and channel-drain junction capacitance, which however, might be significant in the thicker body FinFET device. Therefore, to perform a worst-case comparison of the TFET against the FinFET device, we set the junction capacitances in the latter to zero.

The interconnect parasitic components are shown in **Supplementary Figure 9d** where the contact/via resistance are shown as R_{con}, the magnetic **[20]** inductances are shown as l_{M}, the electrostatic capacitances by c_E, and the interconnect resistance per unit length is r. The parasitics of the interconnect are modeled by a distributed RLC line. The cross-section of the wire has a width of w_{wire} and a height of h_{wire}, which are assumed **[18]** to be 6.375 nm and 12.75 nm respectively. The resistivity of copper (Cu) interconnect is $9 \times 10^{-8}\,\Omega$m, the capacitance is $0.95 \times 10^{-10}\mathrm{F/m}$, while the wire inductance can be assumed to be zero. Connecting each interconnect are two vias on either side which eventually connect to the transistor, whose resistance (R_{via}) is assumed to be 67 Ω. Successive logic blocks in the designed NM circuit have been assumed to be connected by interconnects of equal length, which have been tuned to yield a net interconnect capacitance equivalent to half of the net device capacitance.

<u>Device and Interconnect Capacitance Extraction</u>: The device capacitance of both the 2D-TFET and the LSTP implementations were extracted by simulating the entire circuit without consideration of any interconnects across a range of V_{DD}, with several clock frequencies at each V_{DD}. Evaluation of the average power dissipation in the circuit over the entire duration of its operation, i.e., including both neuron firing and the synaptic weight learning events, gives us a linear dependence of the power consumption with the simulated frequency at a particular V_{DD} following: $P_{\mathrm{total}} = P_{\mathrm{static}} + AF.\,C_{\mathrm{L}}V_{\mathrm{DD}}^2 f$, where P_{total} is the net power dissipation, P_{static} is the net static power dissipation, AF is the activity factor, C_{L} is the net circuit capacitance and f is the frequency of simulation. The slope of this power-frequency curve is $AF.\,C_{\mathrm{L}}V_{\mathrm{DD}}^2$ which yields the net capacitance once V_{DD} and AF are known. **Supplementary Figure 10a** shows the extracted FinFET device capacitance as a function of the simulated V_{DD} which results in an average extracted device capacitance of 46 fF.

In the absence of detailed routing in our implementation, we assume interconnects of equal length connected between adjacent logic blocks, the length of which are estimated by simulations to yield a net interconnect capacitance in the circuit which is equal to half of that of the device capacitance, i.e., 23 fF, in accordance with conventional IC design practices. By extracting the entire circuit capacitance as a function of the interconnect length, a linear relationship between the two is obtained which yields the average capacitance growth as the interconnect length is increased, at ~2.1 fF/100 nm **(Supplementary Figure 10b)**. Therefore, an approximate interconnect length of 1000 nm is chosen to deliver the desired interconnect capacitance of 23 fF, which is half of that of the device capacitance of 46 fF. To

corroborate this, the entire circuit capacitance with 1000 nm interconnect length is then simulated and the average capacitance extracted over a range of V_{DD} which yields an average total capacitance of ~70 fF, thereby confirming the interconnect length (**Supplementary Figures 10c,10d**). With the interconnect length and device capacitance thus established, the TFET and LSTP circuits can now be compared on an even footing. Note that any difference in the capacitance values not only impacts delay/performance (maximum frequency, $f_{\max}$, at same V_{DD}), but also creates differences in the total energy per cycle at the minimum operating point where the switching ($C_{\mathrm{L}}V_{\mathrm{DD}}^2$) and leakage energies are roughly in balance, therefore, it is imperative that the capacitances be correctly accounted for.

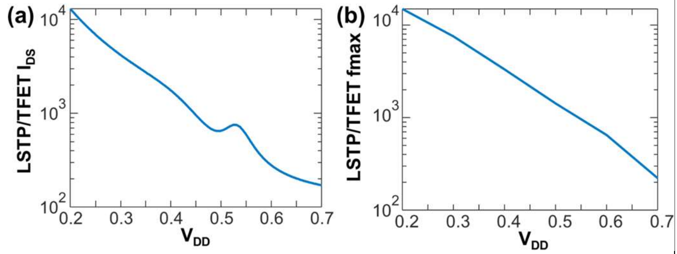

Supplementary Figure 11. MOSFET to TFET I_{DS} and $f_{\max}$ ratio exhibiting identical behavior. (a) Ratio of the I_{DS}-V_{GS} characteristics of the LSTP to the TFET device used in the NM circuit simulations. **(b)** shows the ratio of the $f_{\max}$ of the LSTP and the TFET circuit implementations, showing a behavior equivalent to (a), thereby demonstrating the presence of equal circuit capacitance in the two implementations.

The proof of the equal circuit capacitance in both the TFET and the LSTP circuits can also be observed by comparing the drive current of the two devices against their respective $f_{\max}(\propto I_{\mathrm{DS}}/C_{\mathrm{L}}.V_{\mathrm{DD}})$ as shown in **Supplementary Figure 11**. Since the maximum frequency of operation of a circuit is proportional to the drive current for the same capacitance, hence, the fact that the ratio of the drive current of the LSTP to TFET devices is similar to the ratio of their $f_{\max}$ (**Supplementary Figure 11**) proves that the net capacitance in the two circuits is also equivalent to each other.

<u>Supplementary Note 8:</u> Energy Dissipation per Clock Cycle at Various AFs

The energy dissipation per clock cycle as a function of $f_{\max}$, corresponding to V_{DD} ranging from 0.2V to 0.7V, is plotted at various activity factors. Since the dynamic energy dissipation of the circuit is proportional to the activity factor (*AF*), therefore, as the *AF* is varied, the contribution of the dynamic energy dissipation to the net energy dissipation changes, and the total energy dissipation may solely be determined by the static energy dissipation at very low *AF*. Moreover, since the energy dissipation-V_{DD} plot has different minima at different V_{DD}, therefore, it also helps to ascertain the optimum bias points of operation of the circuit for a particular *AF*.

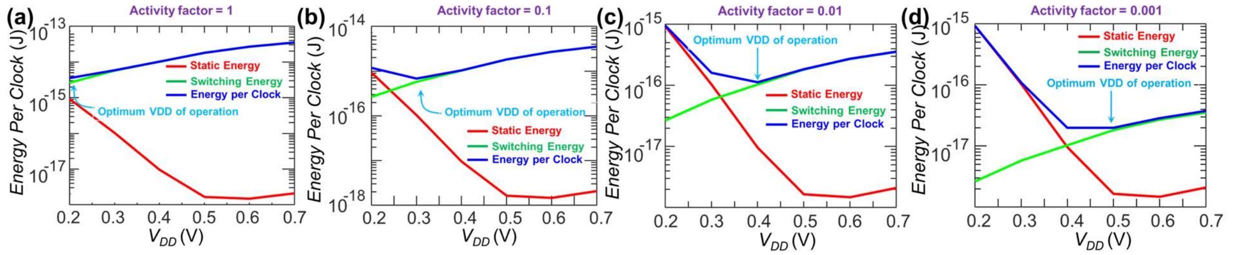

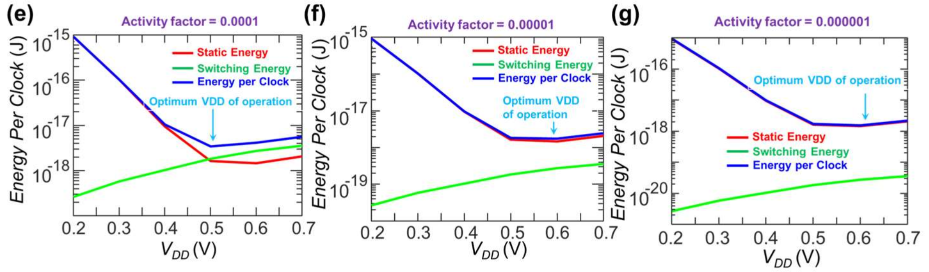

(h)

V_{DD}	E_Switching (J)
0.2V	2.66e-15
0.3V	5.81e-15
0.4V	1.036e-14
0.5V	1.832e-14
0.6V	2.724e-14
0.7V	3.556e-14

Supplementary Figure 12. Energy consumption comparison of the LSTP NM circuit. Energy consumption per clock cycle of the designed NM circuit implemented with LSTP model for a wide range of AFs ranging from **(a)** 1, **(b)** 10^{-1}, **(c)** 10^{-2}, **(d)** 10^{-3}, **(e)** 10^{-4}, **(f)** 10^{-5} and **(g)** 10^{-6}. The energy dissipation was calculated by evaluating the static power and the maximum frequency of operation of the circuit at a particular V_{DD} which yielded the static energy dissipation, while **(h)** the switching energy at each V_{DD} was multiplied with the activity factor to yield the effective dynamic energy dissipation. The V_{DD} which results in the least energy consumption at a particular activity factor, therefore, is the optimal V_{DD} of operation.

<u>*LSTP Circuit:*</u> **Supplementary Figures 12a-12d** show the energy dissipation per clock cycle as the *AF* is decreased from 1 to 10^{-3} respectively, for the LSTP circuit, while **Supplementary Figure 12e-12g** is for *AF* ranging from 10^{-4} to 10^{-6}. **Supplementary Figure 12h** shows a table for the extracted switching energies as a function of the V_{DD}. As seen from **Supplementary Figure 12a-12d**, the static energy is negligible compared to the dynamic/switching energy at

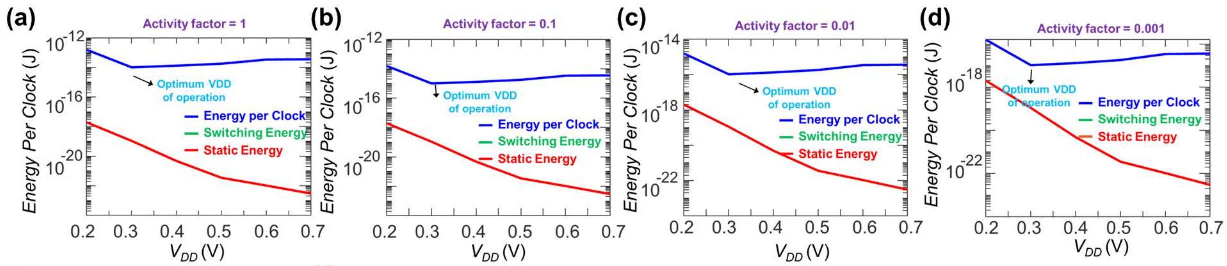

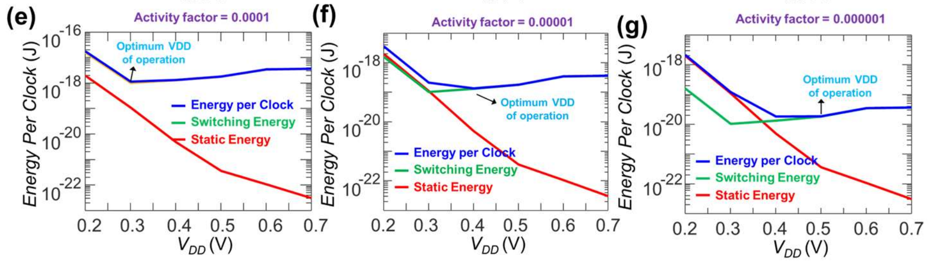

(h)

V_{DD}	E_Switching (J)
0.2V	1.60E-13
0.3V	1.04E-14
0.4V	1.33E-14
0.5V	1.83E-14
0.6V	3.49E-14
0.7V	3.68E-14

Supplementary Figure 13. Energy consumption comparison of the 2D-TFET NM circuit. Energy consumption per clock cycle of the designed NM circuit implemented with TFET model for a wide range of *AF*s ranging from **(a)** 1, **(b)** 10^{-1}, **(c)** 10^{-2}, **(d)** 10^{-3}, **(e)** 10^{-4}, **(f)** 10^{-5} and **(g)** 10^{-6}. The switching energy at each V_{DD} is shown in **(h)**. The inordinately small frequency of operation of the circuit at 0.2V V_{DD} **(Figure 5** of the main article**)** makes the static energy dissipation similar to the switching energy dissipation, thereby making the extraction of the capacitance, and hence, the switching energy, difficult at that bias voltage.

high *AF*s, and therefore, the net energy dissipation in the circuit is dominated by the switching energy. Since the switching energy is $C_{\mathrm{L}}V_{\mathrm{DD}}^2$, therefore, the minimum energy dissipation happens at lowest V_{DD}, and is therefore, the optimum V_{DD} of operation. As the *AF* is reduced however, like in **Supplementary Figure 12e-12g**, the switching energy becomes comparable to the static energy, and the net energy dissipation in the circuit is determined by the contribution of both. Interestingly, the static energy dissipation exhibits a non-linear dependence on the V_{DD} because it is defined as $(P_{\mathrm{static}}/f_{\mathrm{max}})$, and although both P_{static} and f_{max} have a non-linear dependence **(Figure 6** of the main article**)** on V_{DD}, the net static energy dissipation is defined as the ratio of these two, and therefore, can increase if the decrease in f_{max} is faster than the decrease in P_{static}, as is apparent from **Figure 6a-6d** of the main article. This, therefore, moves the optimum V_{DD} of operation to higher V_{DD} at very low *AF*, from 0.2V at *AF* = 1 to 0.6V at *AF* = 10^{-6}. Also, as expected, the switching energy increases with increase in the V_{DD} (**Supplementary Figure 12h**).

2D-TFET Circuit: The energy dissipation per clock cycle was also evaluated for the TFET circuit at various *AF* ranging from 1 to 10^{-6}, as seen in **Supplementary Figure 13a-13g**. As observed from **Supplementary Figure 13a-13e**, the static energy of the TFET is much smaller than that of its switching energy which is determined by the net capacitance of the circuit, and therefore, the net energy dissipation in the circuit is determined by its switching energy for all activity factors ranging from 1 to 10^{-4}. It is only at lower *AF*s **(Supplementary Figures 13f,13g)** that the static energy starts dominating the energy consumption of the circuit, and this too happens at much lower V_{DD} ranges below 0.4V where the much smaller f_{max} causes the static energy to increase by orders of magnitude compared to its value at higher V_{DD}. This, therefore, causes the optimum V_{DD} of operation to shift from lower V_{DD} (0.3V) at higher *AF*s to higher V_{DD} of 0.5V at the lowest *AF* of 10^{-6}. The switching energy of the circuit is extracted at the simulated V_{DD} in **Supplementary Figure 13g**. Since the static energy of the TFET is much smaller than that of the LSTP circuit, and the net circuit energy dissipation is dominated by the static energy at only very low *AF*s, therefore, it is understood that the highest savings in energy dissipation of the TFET over the LSTP implementation are observed at the lowest *AF*.

Supplementary Note 9: Comparison of the Energy Dissipation of the 2D-TFET vs LSTP Implementations

The energy dissipation of the 2D-TFET and the LSTP implementations as a function of V_{DD} for various *AF*s are compared in this section. The simulation assumes that the frequency of the circuit clock at each V_{DD} is equal to its f_{max} for minimizing the static energy dissipation. **Supplementary Figure 14a-14g** compares the energy dissipation of the two implementations as the *AF* is decreased from 1 to 10^{-6}. As seen from **Supplementary Figure 14a-14d** the energy dissipation of both the TFET and the LSTP circuits are quite similar to each other at corresponding V_{DD} because the higher activity factor (*AF*) makes the switching energy the most dominant means of energy dissipation. Since the switching energy is equal to $AF.\,C_{\mathrm{L}}V_{\mathrm{DD}}^2$, therefore, by ensuring equal circuit capacitance (C_{L}), the energy dissipation of

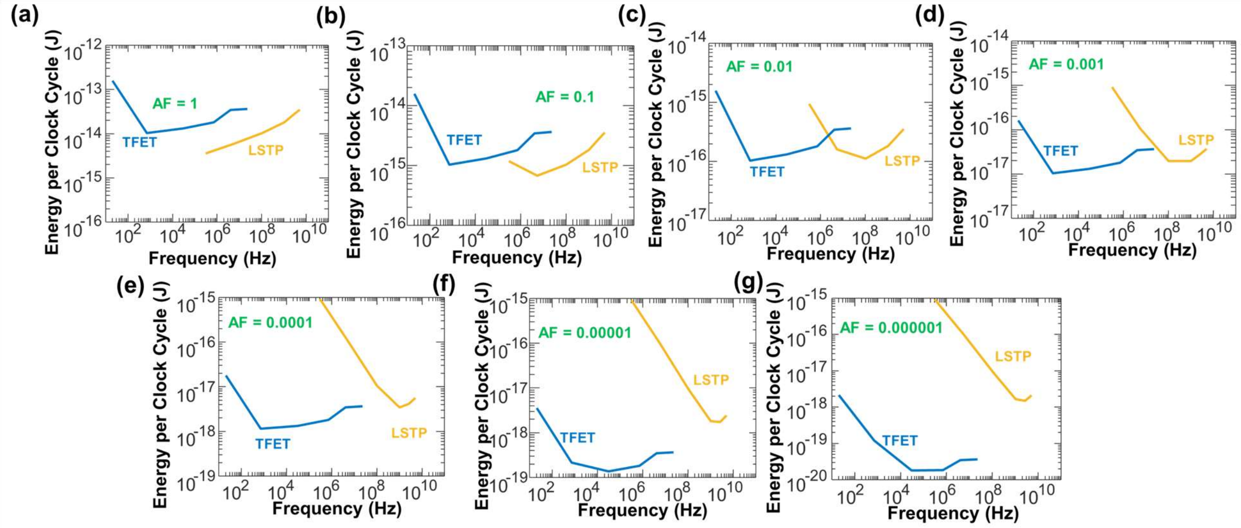

Supplementary Figure 14. Energy consumption comparison of 2D-TFET vs MOSFET. Comparison of the energy dissipation per clock cycle for the TFET and the LSTP implementations as a function of the frequency of operation for various activity factors of **(a)** 1, **(b)** 10^{-1}, **(c)**10^{-2}, **(d)** 10^{-3}, **(e)** 10^{-4}, **(f)** 10^{-5} and **(g)** 10^{-6}. The simulated frequencies correspond to the respective V_{DD} of operation. Highest benefit in the energy dissipation of the TFET implementation over the LSTP implementation is observed at the lowest activity factors. f_{max} of the 2D-TFET also does not go as high as that of the LSTP because of the limited ON-current of the former.

both the TFET and the LSTP implementations can be made equivalent to each other at a particular *AF*. However, as *AF* starts to decrease further (**Supplementary Figure 14e-14g**), the relative contribution of the switching energy to the total energy dissipation starts to decrease, and the net energy dissipation starts getting limited by the static energy. Since the static energy of the TFET is much smaller than that of the LSTP implementation, therefore, the best-case performance of TFET is observed at these lower *AF*s.

Supplementary References

[1]. Pal, A. et al. 2D-Materials enabled next-generation low-energy compute and connectivity. *MRS Bulletin* **46**, 12 (2021).
[2]. Turing, A. Intelligent machinery. 1948. *The Essential Turing* 395 (1969).
[3]. Mead, C. Neuromorphic electronic systems. *Proceedings of the IEEE* **78**, 1629-1636 (1990).
[4]. Hebb, D. O. The organization of behavior: A neuropsychological theory. *Psychology Press*, 2005.
[5]. Maass, W. Networks of spiking neurons: the third generation of neural network models. *Neural networks* **10**, 1659-1671 (1997).
[6]. Pfeiffer, M. & Pfeil, T. Deep learning with spiking neurons: opportunities and challenges. *Frontiers in neuroscience* **12**, 774 (2018).
[7]. Davies, M. et al. Loihi: A neuromorphic manycore processor with on-chip learning. *IEEE Micro* **38**, 82-99 (2018).
[8]. Merolla, P. A. et al. A million spiking-neuron integrated circuit with a scalable communication network and interface. *Science* **345**, 668-673 (2014).
[9]. Cao, W. & Banerjee, K. Is negative capacitance FET a steep-slope logic switch? *Nature Communication* **11**, 1 (2020).
[10]. Marin, E. G., Marian, D., Perucchini, M., Fiori, G. & Iannaccone, G. Lateral heterostructure field-effect transistors based on two-dimensional material stacks with varying thickness and energy filtering source. *ACS Nano* **14**, 1982 (2020).
[11]. Mayer, F., Royer, C. L., Blachier, D., Clavelier, L. & Deleonibus, S. Avalanche breakdown due to 3-D effects in the impact-ionization MOS (I-MOS) on SOI: reliability issues. *IEEE Transactions on Electron Devices* **55**, 1373 (2008).
[12]. Dadgour, H., Hussain, M. M., Cassell, A., Singh, N. & Banerjee, K. Impact of scaling on the performance and reliability degradation of metal-contacts in NEMS devices. *IEEE International Reliability Physics Symposium*, 3D.3.1-3D.3.10 (2011).
[13]. Ajayan, P., Kim, P. & Banerjee, K. Two-dimensional van der Waals materials. *Physics Today* **69**, 38 (2016).
[14]. Cao, W., Kang, J., Sarkar, D., Liu, W. & Banerjee, K. 2D semiconductor FETs—Projections and design for sub-10 nm VLSI. *IEEE Transactions on Electron Devices* **62**, 3459 (2015).
[15]. Cao, W. et al. 2-D layered materials for next-generation electronics: Opportunities and challenges. *IEEE Transactions on Electron Devices* **65**, 4109 (2018).
[16]. Cao, W., Jiang, J., Kang, J., Sarkar, D., Liu, W. & Banerjee, K. Designing band-to-band tunneling field-effect transistors with 2D semiconductors for next-generation low-power VLSI. *IEEE Int. Elec. Dev. Meeting (IEDM)*, 1231-1234 (2015).
[17]. Pal, A., Cao, W. & Banerjee, K. A compact current–voltage model for 2-D-semiconductor-based lateral homo-/hetero-junction tunnel-FETs. *IEEE Transactions on Electron Devices* **67**, 4473-4481 (2020).
[18]. Jiang, J. et al. Intercalation doped multilayer-graphene-nanoribbons for next-generation interconnects. *Nano Letters* **17**, 1482-1488 (2017).
[19]. Clark, L.T. et al. ASAP7: A 7-nm FinFET Predictive Process Design Kit. *Microelectronics Journal* **53**, 105-115 (2016).
[20]. Kang, J. et al. On-chip intercalated-graphene inductors for next-generation radio frequency electronics. *Nature Electronics* **1**, 46-51 (2018).
